# Disseminated Gonococcal Infection Presenting as an Isolated Rash in a Young Patient: A Case Report and Review of Atypical Presentations

**DOI:** 10.7759/cureus.79598

**Published:** 2025-02-24

**Authors:** Mohammed Haroun, Bekure B Siraw, Dinara Salimova, Yonas Gebrecherkos, Tatevik Aloyan

**Affiliations:** 1 Internal Medicine, Ascension Saint Joseph Hospital, Chicago, USA

**Keywords:** ceftriaxone therapy, dgi, gonorrhea, leukocytoclastic vasculitis, methamphetamine, msm, naat, petechial rash, sti

## Abstract

*Neisseria gonorrhoeae *(*N. gonorrhoeae*)is a Gram-negative diplococcus responsible for sexually transmitted infections (STIs) worldwide. While gonorrhea primarily affects mucosal surfaces, disseminated gonococcal infection (DGI) can occur, presenting with polyarthritis, tenosynovitis, and dermatitis. However, atypical presentations can delay diagnosis and treatment. Men who have sex with men (MSM) are commonly affected by gonorrhea, necessitating vigilance for DGI in this population.

A 24-year-old MSM with a history of previously treated gonorrhea presented with a petechial rash on the lower extremities and mild, painless foot swelling without systemic symptoms or joint involvement. The patient had engaged in multiple unprotected sexual encounters in the preceding month. Physical examination revealed a non-itchy, non-blanching rash, most prominent on the dorsum of the feet and sparing the soles. Laboratory findings showed leukocytosis and pyuria, while bacterial cultures remained negative. Nucleic acid amplification testing (NAAT) from urine was positive for *N. gonorrhoeae*, confirming the diagnosis. Given the absence of joint symptoms, purulent arthritis was considered unlikely, and leukocytoclastic vasculitis (LCV) was a differential diagnosis. However, the patient demonstrated significant improvement with ceftriaxone and doxycycline, supporting DGI as the primary diagnosis. He completed a seven-day course of ceftriaxone and oral doxycycline with full resolution of symptoms at follow-up.

This case highlights an unusual presentation of DGI with isolated dermatologic manifestations and no tenosynovitis or arthritis. Methamphetamine use, previously linked to increased susceptibility to DGI, was noted in this patient. Negative blood and mucosal cultures emphasize the role of NAAT in diagnosing atypical DGI. Comparative case reviews demonstrate a wide spectrum of DGI presentations, reinforcing the need for early recognition.

Clinicians should maintain a high index of suspicion for DGI, particularly in at-risk populations such as MSM, even in the absence of classic symptoms. Prompt diagnosis using NAAT and early antibiotic initiation are crucial for preventing complications. This case underscores the need for heightened awareness of atypical presentations to optimize patient outcomes.

## Introduction

*Neisseria gonorrhoeae* (*N. gonorrhoeae*) is a Gram-negative diplococcus and a major cause of sexually transmitted infections (STIs) worldwide. It primarily affects the mucous membranes of the urethra, cervix, pharynx, and rectum, leading to localized infections that often present with urethritis, cervicitis, pharyngitis, and proctitis. In some cases, however, *N. gonorrhoeae* can invade the bloodstream and disseminate to distant tissues, leading to disseminated gonococcal infection (DGI). This condition occurs in approximately 0.5% to 3% of gonorrhea infections [1-3].

Disseminated gonococcal infection manifests in two forms. The more prevalent form is characterized by a triad of migratory polyarthritis, skin lesions, and tenosynovitis, whereas the less common form presents as purulent arthritis [4]. The pathogenesis of DGI involves the hematogenous spread of *N. gonorrhoeae* from a primary mucosal site (e.g., urethra, pharynx, or rectum) to distant tissues facilitated by immune evasion mechanisms unique to the pathogen. Risk factors for DGI include female sex, pregnancy, multiple sexual partners, as well as different forms of immunodeficiencies, including, but not limited to, HIV, alcohol abuse, complement deficiencies, and others [5].

In the United States, gonorrhea remains a significant public health concern, with increasing rates observed over the past decade. According to the Centers for Disease Control and Prevention (CDC), over 700,000 cases of gonorrhea are reported annually, with men having sex with men (MSM) disproportionately affected [6]. This population accounts for an estimated one-third of reported gonorrhea cases, a trend attributed to factors such as high-risk sexual behaviors, inconsistent condom use, and barriers to healthcare access in certain communities [6]. The increasing prevalence of gonorrhea in MSM has raised concerns about the emergence of antimicrobial resistance, further emphasizing the need for prompt diagnosis and treatment of DGI to prevent complications [6].

Given the rising prevalence of gonorrhea among MSM populations and the potential for subclinical infection [6], clinicians must remain vigilant for unusual presentations of DGI. This case highlights one such atypical presentation, underscoring the need for a high index of suspicion in at-risk groups and the importance of early recognition to prevent complications.

## Case presentation

A 24-year-old male patient who identifies as a homosexual with a history of previously treated gonorrhea presented to the emergency department with a complaint of bilateral foot rash and slight painless right foot swelling. The patient reported that the rash began one day prior to admission. He speculated that wearing a new pair of shoes without socks might have caused it. He additionally complained of penile discharge that began one week prior to his presentation. He disclosed that he completed a course of antibiotics for gonorrhea, urethritis, and chlamydia approximately three months prior to these presentations with the resolution of his symptoms afterward, though he could not recall the specific antibiotic used. Over the past month, he reported having between one and five sexual partners and noted inconsistent condom use. Of note, he reported a history of regular crystal methamphetamine use, with his last use occurring approximately one hour prior to arrival at the hospital. The patient denied systemic symptoms such as joint pain, shortness of breath, chest pain, itching, fever, headache, neck pain, chills, abdominal pain, or vomiting. He also denied regular medication use, recent travel history, viral illnesses, hematuria, dysuria, and a history of being immunocompromised.

**Figure 1:**
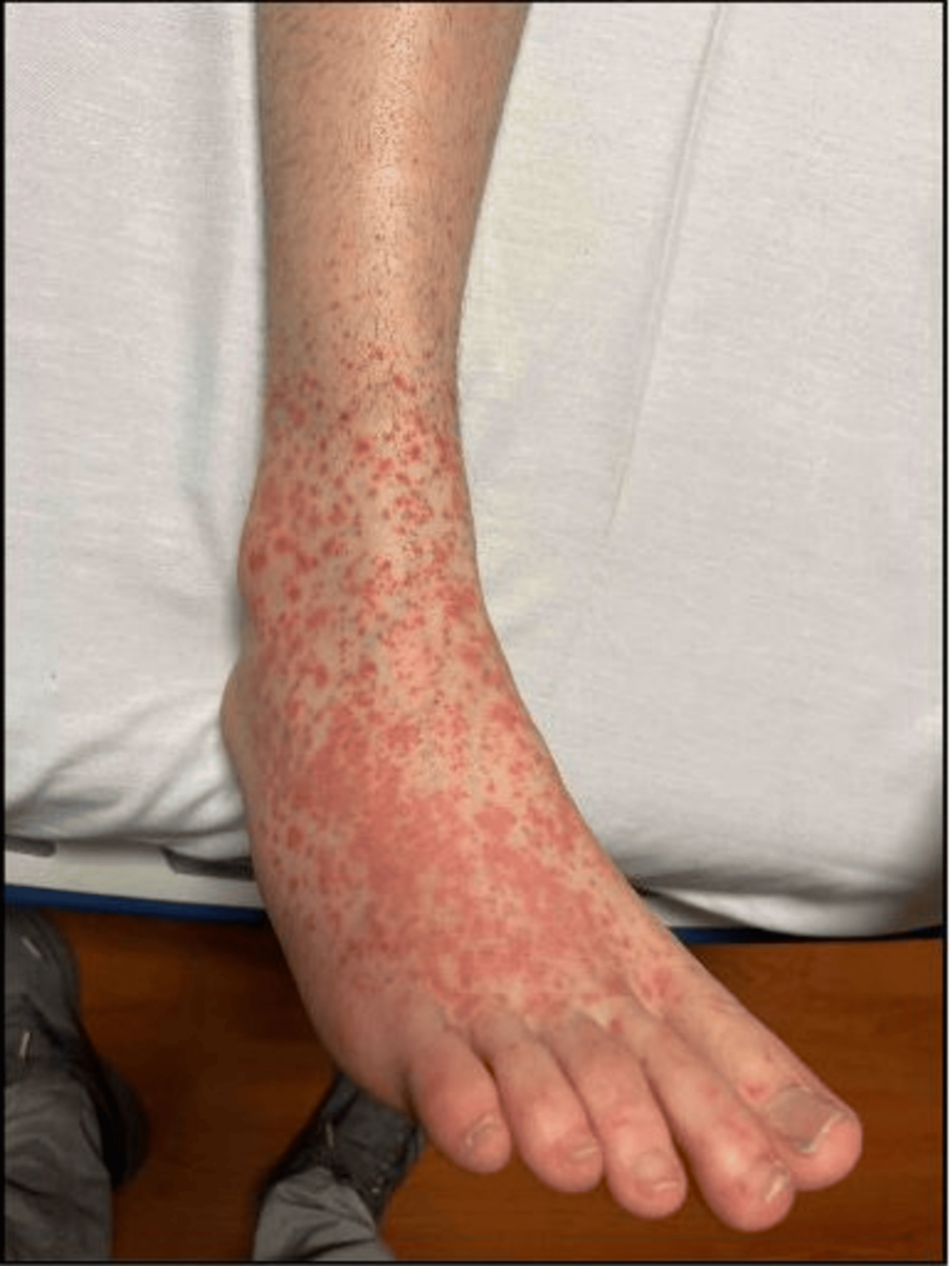
Non-palpable, Non-tender, and Non-blanching Petechiae Over the Dorsum of the Right Foot

**Figure 2:**
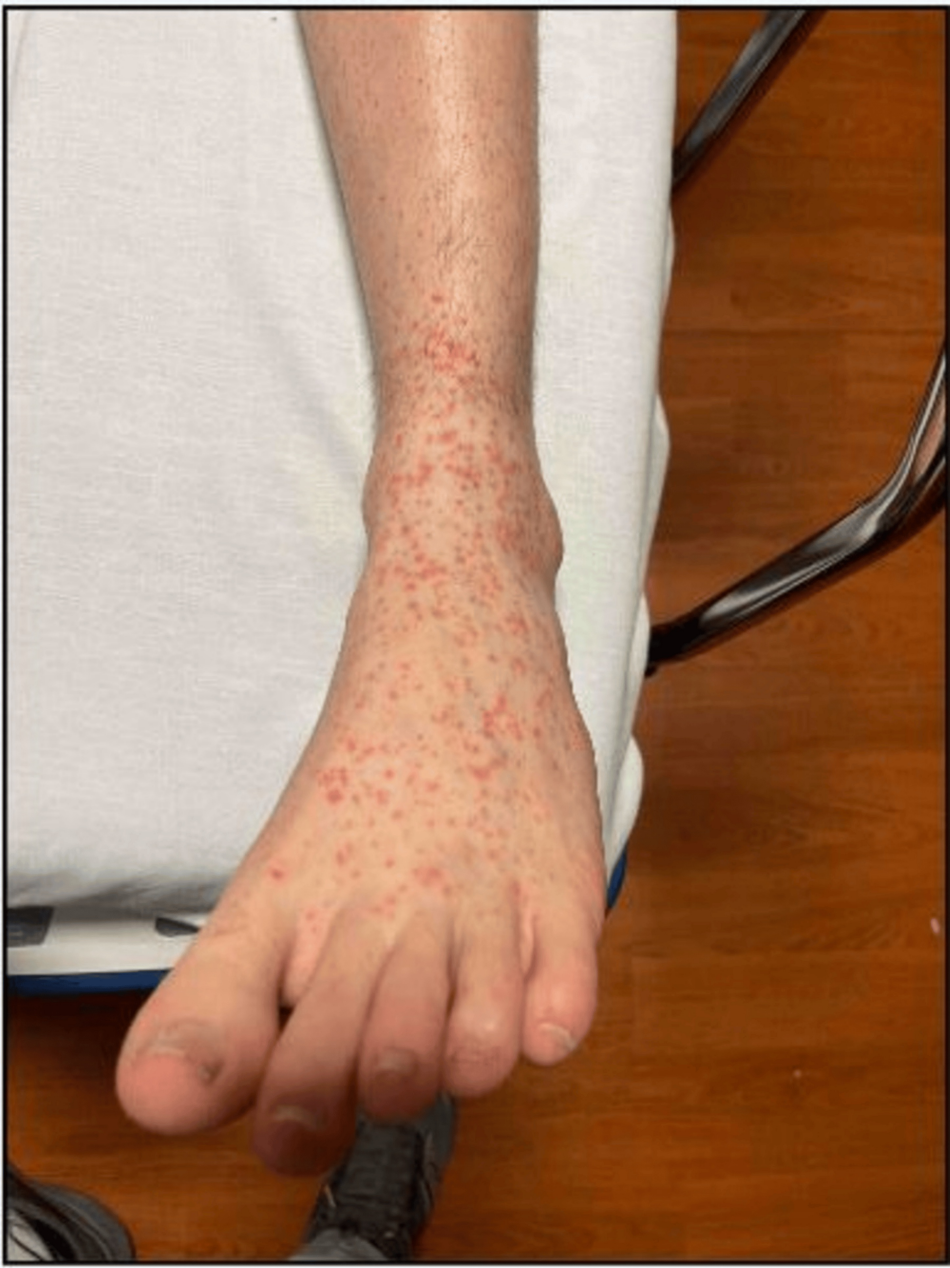
Non-palpable, Non-tender, and Non-blanching Petechiae on the Dorsum of the Left Foot

The patient was afebrile, not tachycardic, with normal oxygen saturation on room air and a blood pressure of 122/68 mmHg. Physical examination was remarkable for a petechial, non-itchy, non-blanching, non-tender, and non-raised rash on the bilateral lower extremities. It was most prominent on the dorsum of the feet, sparing the soles, with satellite lesions on the shins and associated mild, painless swelling of the right foot (Figures 1, 2). There were no genital lesions, buttock lesions, splinter or conjunctival hemorrhages, Janeway lesions, Osler’s nodes, or heart murmurs. The rest of the systems were unremarkable on examination. 

Extensive laboratory workup is shown in Table 1, which was remarkable for slight leukocytosis, sterile pyuria, and a positive urine nucleic acid amplification test (NAAT) for *N. gonorrhoeae*.

**Table 1:** The Patient’s Lab Workup Results

Test	Result	Reference Range
White Blood Cell (WBC) Count	13.3 k/mm3	4.0 - 11.0 k/mm3
Platelet Count	438 k/mm3	150 - 450 k/mm3
Complete Metabolic Panel (CMP)	Unremarkable	Normal
Prothrombin Time (PT)/International Normalised Ratio (INR) (Day of Admission)	14.4 sec / 1.3	10 - 13.1 sec / 0.9 - 1.1
Urinalysis: Appearance	Cloudy	Clear
Urinalysis: Protein	30 mg/dL	<10 mg/dL
Urinalysis: WBCs/High-Power Field (HPF)	>100 WBCs/HPF	<5 WBCs/HPF
Urine Toxicology	Positive for Amphetamines	Negative
Bacterial Blood Cultures	No Growth	No Growth
Urine Cultures	No Growth	No Growth
Erythrocyte Sedimentation Rate (ESR)	12 mm/hr	0 - 20 mm/hr
Complement Levels (C3, C4)	Within Normal Limits	Within Normal Limits
Antinuclear Antibody (ANA)	Negative	Negative
Nucleic Acid Amplification Test (NAAT) for *Neisseria gonorrhoeae* (urine)	Positive	Negative
NAAT for *Chlamydia trachomatis* (urine)	Negative	Negative
NAAT for *Trichomonas *(urine)	Negative	Negative
Rapid Plasma Reagin (RPR) (Syphilis Test)	Non-Reactive	Non-reactive
HIV Screening Test	Non-Reactive	Non-reactive
Hepatitis Panel (A, B and C)	Non-Reactive	Non-reactive

Based on the clinical presentation, laboratory findings with a positive gonococcal NAAT, and the patient's history, a diagnosis of DGI versus leukocytoclastic vasculitis (LCV) was considered. The patient was started on intravenous ceftriaxone 2 g once daily and oral doxycycline 100 mg twice daily for coverage of chlamydia. The patient showed an interval improvement of the rash within the next three days of the hospital stay, further supporting the DGI diagnosis. On day three of admission, the patient was discharged on a seven-day course of oral doxycycline 100 mg twice daily for post-exposure prophylaxis and coverage of chlamydia and to finish a course of seven days of IV ceftriaxone 2 g daily in an outpatient infusion center. The patient was followed up one week after finishing his antibiotic courses, and his rash resolved completely.

## Discussion

This case highlights an atypical presentation of DGI in a young MSM patient. While one of the classic presentations of DGI is with the triad of tenosynovitis, dermatitis, and migratory polyarthritis, this case deviates significantly by presenting solely with a petechial rash and painless foot swelling without systemic symptoms or joint involvement. Although studies have shown a disproportionately high prevalence of gonorrhea among MSM [6], DGI remains uncommon in this population [7-9].

A 2008 case reported a homosexual man with classic DGI symptoms, including fever, arthralgia, tenosynovitis, and necrotic papules. Diagnosis relied on NAAT of a skin lesion due to negative blood and mucosal site cultures. Similarly, our case involved an MSM man with an atypical presentation of petechial rash and foot swelling without systemic symptoms, and cultures were also negative. Both cases highlight the clinical variability of DGI and the critical role of NAAT in diagnosis when cultures are inconclusive [10]. There is some evidence that *N. gonorrhoeae* strains predisposing to DGI are vancomycin sensitive, which is found in the selective culture media to isolate N. gonorrhea, leading to false-negative results [11].

Tavares et al. described a case of DGI in a 35-year-old male patient presenting with fever, hemorrhagic vesicles, and polyarthralgia in the lack of microbiological confirmation of the infection [12]. In contrast, our patient lacked systemic symptoms but had microbiological confirmation via NAAT, highlighting the diagnostic value of molecular testing in atypical presentations.

Blank et al. described a case of a 36-year-old woman presenting with a petechial rash and bilateral tenosynovitis [13]. This patient’s significant joint involvement contrasts with our case, where no joint symptoms were present. Both cases, however, involved dermatologic findings, positive gonococcal NAAT, and negative blood cultures, emphasizing the importance of recognizing skin manifestations in diagnosing DGI. Furthermore, adherence to prolonged antibiotic therapy was crucial for managing both cases, demonstrating the importance of appropriate and timely treatment.

Baker et al. reported a case of a 22-year-old woman presenting with petechial rashes, chills, and severe joint pain [14]. Diagnosis in this case relied on histopathological findings of septic vasculitis with Gram-negative diplococci. This case illustrates the variability in diagnostic approaches necessary to confirm DGI across its different clinical manifestations.

Tailor et. al. described another atypical and rare presentation of DGI [15], in which a 27-year-old man presented with exertional chest pain, dyspnea, unintentional weight loss, malaise, joint pains, and desquamative rash on both hands. In contrast to our case, this patient was febrile, tachycardic, had a systolic murmur on examination, had a positive blood culture for *N. gonorrhoeae*, and his echocardiography was suggestive of infective endocarditis (IE) with an aortic root abscess. Both patients lacked other stigmata of IE like Janeway lesions, Osler’s nodes, splinter, and conjunctival hemorrhages. This reflects the variability of DGI presentation and the importance of keeping a high suspicion index for diagnosis and prompt treatment to prevent drastic complications.

Behavioral risk factors also play a significant role in the development of DGI. The rise in DGI cases in California between July 2020 and July 2021 revealed that approximately one-third of patients with DGI reported methamphetamine use [7]. Methamphetamine-induced immune suppression may impair mucosal defenses, by disrupting innate immunity through inhibiting phagocytosis, and adaptive immunity by disrupting T-cell response to infection [16], facilitating the dissemination of *N. gonorrhoeae*. In our case, the patient reported regular methamphetamine use, which may have contributed to the development of DGI. This association emphasizes the importance of addressing substance use as part of a comprehensive evaluation and management strategy in at-risk populations. 

Emerging evidence suggests that postexposure prophylaxis with doxycycline could reduce the incidence of bacterial sexually transmitted infections, including gonorrhea, in high-risk populations such as MSMs [17]. While promising, further studies are required to determine its effectiveness in preventing complications like DGI. 

Additionally, the diagnosis of LCV was also considered, as *N. gonorrhoeae* is one of the infectious causes of LCV [18]. A skin biopsy was not needed for this patient, as his rash and swelling improved with antibiotic therapy without the need for steroids.

## Conclusions

This case emphasizes the importance of maintaining a high index of clinical suspicion for DGI, particularly in at-risk populations such as MSM. Atypical presentations without systemic symptoms can lead to diagnostic delays, increasing the risk of complications. Prompt recognition and timely initiation of appropriate antibiotics, such as ceftriaxone and doxycycline, are essential to achieve optimal patient outcomes and prevent serious complications like endocarditis and meningitis.
